# Use of oral health care services in Peru: trends of socio-economic inequalities before and after the implementation of Universal Health Assurance

**DOI:** 10.1186/s12903-019-0731-7

**Published:** 2019-03-07

**Authors:** Akram Hernández-Vásquez, Guido Bendezu-Quispe, Diego Azañedo, Marilina Santero

**Affiliations:** 1 Universidad San Ignacio de Loyola, Centro de Excelencia en Estudios Económicos y Sociales en Salud, Lima, Peru; 2 Universidad San Ignacio de Loyola, Unidad de Investigación para la Generación y Síntesis de Evidencias en Salud, Lima, Peru; 3 Universidad Católica los Ángeles de Chimbote, Instituto de Investigación, Chimbote, Peru; 4 Universidad de Buenos Aires, Buenos Aires, Argentina; 5 Instituto de Efectividad Clínica y Sanitaria (IECS), Buenos Aires, Argentina

**Keywords:** Oral health, Healthcare disparities, Health services misuse, Cross-Sectional Studies, Surveys and Questionnaires, Peru (source: MeSH NLM)

## Abstract

**Background:**

Oral health inequalities are profound worldwide. Despite major improvements in oral health, inequalities exist for many racial and ethnic groups, by socioeconomic status, gender, age, and geographic location. Therefore, the purpose of this study was to investigate trends of socio-economic inequalities in access to oral health services in Peru before and after the implementation of Universal Health Assurance (AUS).

**Methods:**

Analytical cross-sectional study based on the National Household Survey on Living Conditions and Poverty (ENAHO) 2004, 2008, 2010 and 2017. Two periods were defined before and after the AUS Law (2009). Use of oral health services was the dependent variable, for the general population and according to ages, the area of residence, and natural region. Measurements of inequality in the use of health services were made based on the concentration curves (CC), dominance test and concentration index (CI).

**Results:**

We included a number of 85,436 (2004), 88,673 (2008), 87,074 (2010) and 124,142 (2017) participants. The proportion of people who used oral health services was 8.4% (2014), 10.1% (2008), 10.6% (2010) and 10.4% (2017). Use of oral health services showed an increase in different age groups, urban and rural areas, and natural regions of residence during the study period. The CC were distributed below the line of equality, indicating an inequality of use of oral health services, in favor of the richest groups and dominance of the CC in 2017 over the previous years. Changes in the CI were statistically significant for < 5 years and in the rural area, and for the period 2010-2017 they were also significant in the general population, children aged 5-17 years, urban area, and Andean and Jungle regions, which indicates a reduction in the concentration of use of these services in these groups.

**Conclusions:**

The use of oral health services in Peru increased and inequality decreased in the period 2004-2017, coinciding with the implementation of the AUS. However, the use of these services continue having a distribution in favor of the richest populations. It requires the introduction of new strategies and oral health programs in the Peruvian population, with the aim of closing the gap currently mediated by the economic possibilities.

## Background

Oral Health is a determinant factor for good health and optimal quality of life, based on the health burden that it represents and its relationship with the presence of different chronic conditions [1]. Worldwide, 60-90% of schoolchildren and almost 100% of adults face tooth decay problems among others that encompass the oral cavity [2]. The oral health burden of disease is presented heterogeneously around the world, with the highest burden in low and middle-income countries due to inadequate fluoride-based interventions and low access to primary oral health care services [2].

In Peru, several inequalities in health have been described by natural or administrative regions, rural and urban residency settings, age groups, income quintiles, inter alia. These inequalities are related to the fragmented health system and the low investment that has been made [3]. Oral health is one of the most neglected aspects of the Peruvian health system, with a minimal dental services utilization despite a high prevalence of oro-dental pathologies [4–6].

Peruvian 2014 Demographic and Health Survey (DHS) showed a rather discouraging outlook concerning the oral health services utilization, where only 22% of the elderly attended any dental health service [7]. Furthermore, only 27% of children under 12 years used any dental service in the previous 6 months prior to the survey [8]. Percentage of utilization in both groups are low taking in account the at least 2 visits per year internationally accepted recommendation [9]. Additionally, studies show important inequalities in the oral health services utilization by many other characteristics like type of health insurance, residency region and urban or rural setting [7, 8]. These findings show a problem of great magnitude that needs to be analyzed and addressed by decision makers and health researchers to shorten the inequality gaps in Peru.

Until 2008, 40% of the Peruvian population was not affiliated with a health service [10]. With the deployment of the Universal Health Assurance (AUS, acronym in Spanish) in 2009, the Peruvian State included a package of essential preventive and recuperative services to be provided to the entire population, including dental care [11–13]. The operation of this law eliminates or minimizes out-of-pocket expenses or co-payments [14, 13]. Many international experiences, such as China and Chile [15, 16], showed a reduction on inequality in oral health services utilization after the launch of a universal insurance plan. Considering that one of the main goals of AUS is to shorten the health services’ economic access gaps – and with it the health inequalities –, the goal of this study was to analyze trends of socio-economic inequalities in access to oral healh services before and after the implementation of AUS in Peru.

## Methods

### Study design and data sources

An analytical cross-sectional study was performed based on 2004, 2008, 2010 and 2017 Peruvian National Household Survey on Living Conditions and Poverty (ENAHO, acronym in Spanish). These surveys have been executed by the National Statistics and Informatics Institute (INEI) and their study population is the set of all private dwellings and their occupants living in rural and urban areas of the country.

ENAHO is a yearly cross-sectional survey which uses a probabilistic, of areas**,** stratified, multistage and department-independent sampling. It has national, departmental, natural region and urban-rural representativeness in order to gather information about the living conditions of the Peruvian population. For this study, we selected the years 2004 and 2008 surveys because they are the first and the last survey conducted before the AUS in Peru. It permits comparison in a frame period before the AUS program. In Peru, the AUS program was launched in 2009. For the period after AUS, we selected 2010 and 2017 for the same reason (to compare the first and the last available ENAHO survey data for this period).

### Study variables

The dependent variable of this study is oral health services utilization (p414_06). This self-report variable was built upon people’s answers of having been attended in any oral health service 3 months prior to the survey. The independent variable is the monthly per capita expenditure obtained by dividing the total household expenditure by the number of household members.

Other variables of interest are: 1) General Population: defined as the totality of the Peruvian population; 2) Life stages: including age groups of children less than 5 years old, children between 5 and 17 years old, adults (18–59 year old) and elderly (60 years old or more); 3) Quintile: included five per capita expenditure quintiles (each quintile contains 20% of the total population: quintile 1 is poorest, and quintile 5 is richest); 4) Residency area: defined as habiting rural areas (characterized as being isolated, with low services provision and high levels of poverty), or urban areas; 5) Natural region: this includes the Coast, the Andean, and the Jungle. The coast region is extended along the Pacific Ocean and it contains most of the wealthiest and most developed cities of Peru, including Lima, the capital of the country. The Andean region lies in the Andean zone and has been historically characterized for having high poverty rates and low access to health services due to geographical reasons and short supply. Finally, the Jungle region corresponds to amazon geography with several socioeconomic shortages such as the Andean region.

### Data analysis

Databases included for each survey year were obtained through the Calculated Variables and Health modules in the Microdata section located in the INEI web portal (http://iinei.inei.gob.pe/microdatos/). These databases were integrated through the *append* and *merge* commands.

Through the dental health services utilization weighted means, a descriptive statistical analysis was performed for each year and per capita expenditure quintile. All analyses were estimated with sampling weights and the complex survey design of the ENAHO.

Health services utilization inequality measurement was estimated based on the concentration curves (CC) and concentration indexes (CI), broadly discussed by O’Donnel et al. [17]. In our study, the CCs describe the relationship between population’s per capita expenditure cumulative percentage and the cumulative percentage of dental health services utilization regarding the diagonal equity line. Inequality is estimated according to the curve’s concavity, i.e. the further the CC moves away from the equity line, the bigger the inequality is. Measuring inequality by means of the CCs would show greater percentage of use of oral health services for the population with the highest expenditure levels when it is below the equity line, and, inversely, greater percentage of use of oral health services for the population with the lowest expenditure when the curve is above the equity line [18]. Significant statistical differences between CCs were assessed with the dominance test using the multiple comparison approach (MCA) method. MCA is a decision rule in which only a significant difference is required to establish dominance between multiple quantile comparison points. Further details, test explanation and performed codes can be found in O’Donnel et al. methodological paper [17]. CI is a coefficient whose values lie between − 1 and 1, in which zero means complete equality, a positive value appears when the studied variable is concentrated in the richer population and a negative value when this variable is concentrated in the poorer population. Data management and analysis were performed using the software Stata® (Stata Corporation, College Station, Texas, USA).

### Ethical considerations

This study did not require institutional review board ethical approval because it analyses public domain secondary aggregated data that cannot be used to identify the surveyed participants. ENAHO is a national survey conducted by the INEI. Because this survey is conducted by a governmental institution to generate indicators of interest to measure a country’s development, it is not required a previous approval of the participants to consent their participation.

## Results

A total of 385,298 participants were included in the ENAHO survey data analysis (Table 1). Study participants included were 85,436 (2004), 88,673 (2008), 87,074 (2010) and 124,142 (2017). Participation rates in the ENAHO were as following: ENAHO 2004: 98.8%, ENAHO 2008: 98.9%, ENAHO 2010: 98.8%, and ENAHO 2017: 99.4%. A population aging phenomenon is observed during the study period based on the diminishing of the 5 years or younger population and the increase of the 60 years or older population that exceeds 10%. In 2004, 70.2% inhabited urban areas and this same measurement was 77.6% in 2017. Relating to natural region precedence, in 2004, 53.0% inhabited the Coast region, 34.0% the Andean region and 13.0% the Jungle regions. In 2017, the coastal population represented 55.5% of the Peruvian population (increase in 2.5% from 2004), and there was a reduction in the Andean (31.7%) and jungle (12.8%) percentage of population.

**Table 1:** Background characteristics of respondents, Peru 2004–2017

Characteristic	2004		2008		2010		2017	
	Weighted percent	Unweighted number	Weighted percent	Unweighted number	Weighted percent	Unweighted number	Weighted percent	Unweighted number
Total	100	85,436	100	88,673	100	87,047	100	124,142
Gender								
Men	49.8	42,749	49.1	44,069	49.2	43,075	48.8	60,942
Women	50.2	42,687	50.9	44,604	50.8	43,972	51.2	63,200
Age group								
Under-five children	8.9	7983	9.0	8477	8.5	7812	7.6	9783
Children aged 5–17 years	29.1	26,212	26.7	25,284	26.0	24,339	23.7	31,048
Adults aged 18–59 years	52.2	43,287	53.4	45,793	53.6	45,129	54.2	64,922
Adults aged 60 years or older	9.8	7951	10.9	9119	11.9	9767	14.5	18,389
Place of residence								
Urban	70.2	49,224	72.6	52,935	73.7	51,560	77.6	78,458
Rural	29.8	36,212	27.4	35,738	26.3	35,487	22.4	45,684
Natural region								
Coast	53.0	33,063	53.6	32,810	54.0	32,357	55.5	52,419
Andean	34.0	33,169	33.3	35,463	33.0	34,693	31.7	44,113
Jungle	13.0	19,204	13.1	20,400	13.0	19,997	12.8	27,610

Weighted percentages are according to sampling especifications of ENAHO by year

Table 2 shows that during 2004, 8.4% of the population attended to any dental health services 3 months before the survey, being this proportion 10.1% (2008), 10.6% (2010) and 10.4% (2017) for the following years. This increase is statistically significant between 2004 and 2017 (*p* < 0.001). Between 2010 and 2017 a decrease is described for the 18–59-year-old population (*p* < 0.001), urban area (*p* < 0.001) and coast region (*p* < 0.001). On the other side, a sustained increase in health services utilization is described in rural areas (*p* < 0.001), Andean region (*p* < 0.001), Jungle region (*p* < 0.001) and 5-year-old or younger (*p* < 0.001), 5 to 17-year-old (*p* < 0.001) and older than 60 years (*p* < 0.001) age groups. According to per capita expenditure quintiles, fluctuating values during the study period were found. Prior to the AUS implementation, only the 5th quintile showed a services utilization increase (from 17.5% to 19.2%) between 2004 and 2008, while after the AUS implementation, the utilization percentage declined among all quintiles between 2010 and 2017, being the biggest decline in the 5th quintile (from 18.5% to 14.6%).

**Table 2:** Utilization of dental services by quintiles of expenditure per capita, Peru 2004–2017

Year/Quintile of expenditure per capita	Percentage of population with utilization of dental services									
	General population	Under-five children	Children aged 5–17 years	Adults aged 18–59 years	Adults aged 60 years or older	Urban	Rural	Coast	Andean	Jungle
2004	8.4	2.4	8.8	9.5	6.4	10.0	4.6	9.3	7.6	6.8
Quintile I (poorest)	4.3	0.7	5.2	5.1	1.9	5.7	3.5	4.3	4.3	4.3
Quintile II	7.8	2.4	9.1	8.5	4.6	7.8	8.2	6.6	10.6	8.4
Quintile III	10.7	5.7	11.7	11.2	8.7	10.7	10.6	9.8	13.5	12.1
Quintile IV	15.0	7.3	20.0	15.4	9.5	15.1	13.1	14.5	19.3	12.1
Quintile V (richest)	17.5	8.0	23.4	17.5	14.1	17.5	19.1	16.5	25.2	17.1
2008	10.1	3.9	10.5	11.7	6.5	12.0	5.2	11.4	9.1	7.3
Quintile I (poorest)	4.1	1.9	4.9	4.7	1.3	5.0	3.8	4.0	4.2	3.6
Quintile II	6.8	2.8	8.1	7.5	3.6	7.2	6.0	6.1	8.2	5.9
Quintile III	10.2	4.5	12.2	11.2	5.0	10.4	8.8	9.5	12.8	9.4
Quintile IV	13.3	6.1	15.3	14.3	8.5	13.4	11.5	12.7	17.2	10.7
Quintile V (richest)	19.2	9.6	22.4	20.6	13.1	19.2	18.0	18.4	24.2	17.7
2010	10.6	4.7	12.0	11.7	6.8	12.3	5.9	11.6	9.7	8.6
Quintile I (poorest)	4.4	1.1	5.8	4.6	2.3	5.2	4.2	3.5	4.8	3.5
Quintile II	6.8	2.5	8.1	7.3	4.6	7.1	6.4	5.9	7.7	6.8
Quintile III	9.9	6.3	12.8	10.0	5.4	10.0	9.4	9.1	11.9	9.5
Quintile IV	12.7	7.2	16.3	13.1	7.2	12.9	9.7	11.9	15.8	12.1
Quintile V (richest)	18.5	9.8	23.9	19.5	11.8	18.5	16.9	17.7	22.0	20.2
2017	10.4	6.6	12.8	10.7	7.7	11.5	6.7	10.9	10.2	9.1
Quintile I (poorest)	3.7	1.8	5.2	3.4	1.8	3.1	3.8	2.4	4.1	3.0
Quintile II	6.3	4.2	9.1	5.6	3.0	6.9	5.9	3.9	7.2	5.7
Quintile III	7.5	5.7	11.0	6.6	4.4	7.5	7.5	6.1	8.9	7.8
Quintile IV	10.1	6.7	13.2	10.2	6.2	10.3	8.8	9.5	11.8	10.2
Quintile V (richest)	14.6	10.9	18.8	14.7	11.4	14.6	12.6	13.9	17.5	15.3

**Fig. 1:**
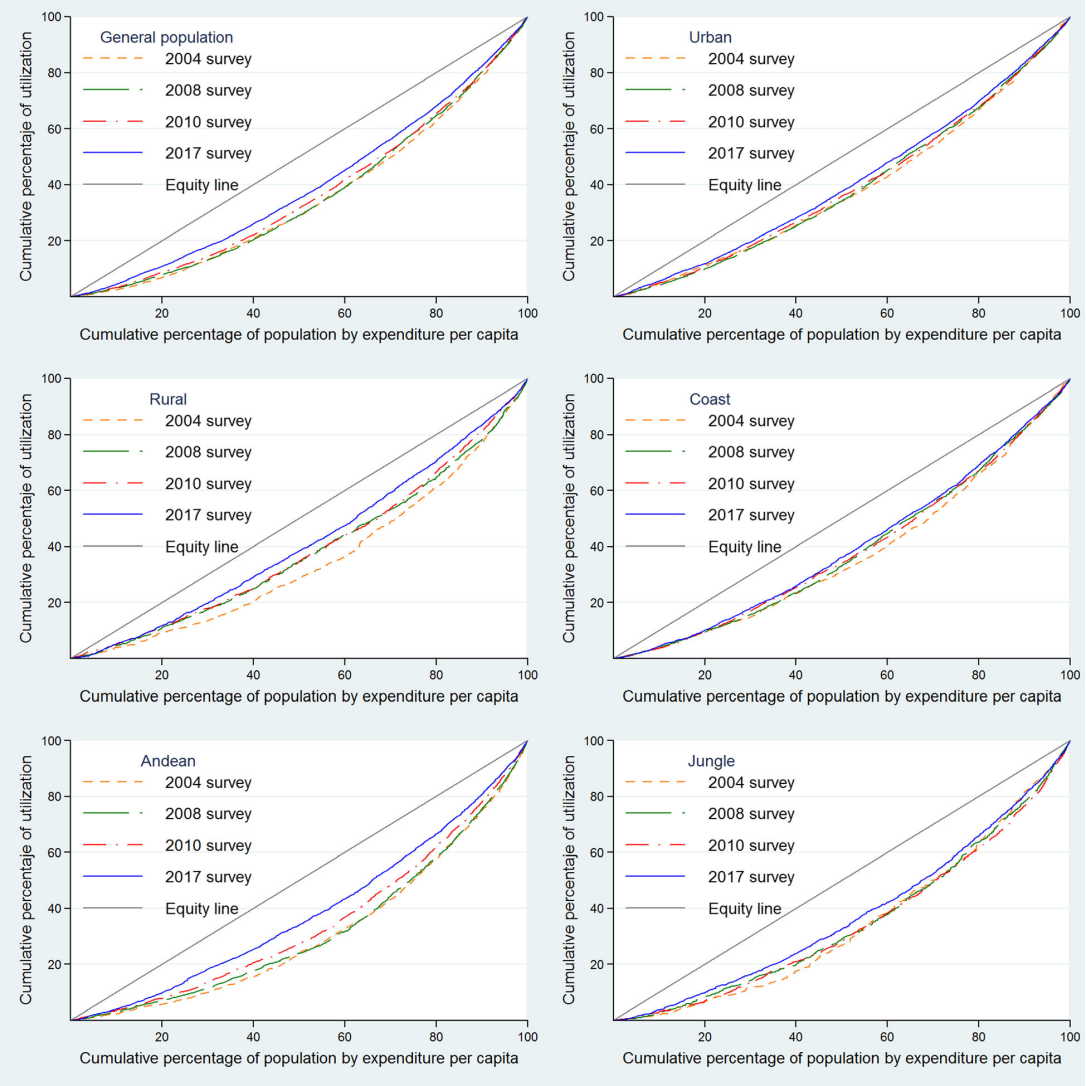
Concentrations curves for access to oral health services by the general population, residency area and natural region. Peru, 2004, 2008, 2010, and 2017**

**Fig. 2:**
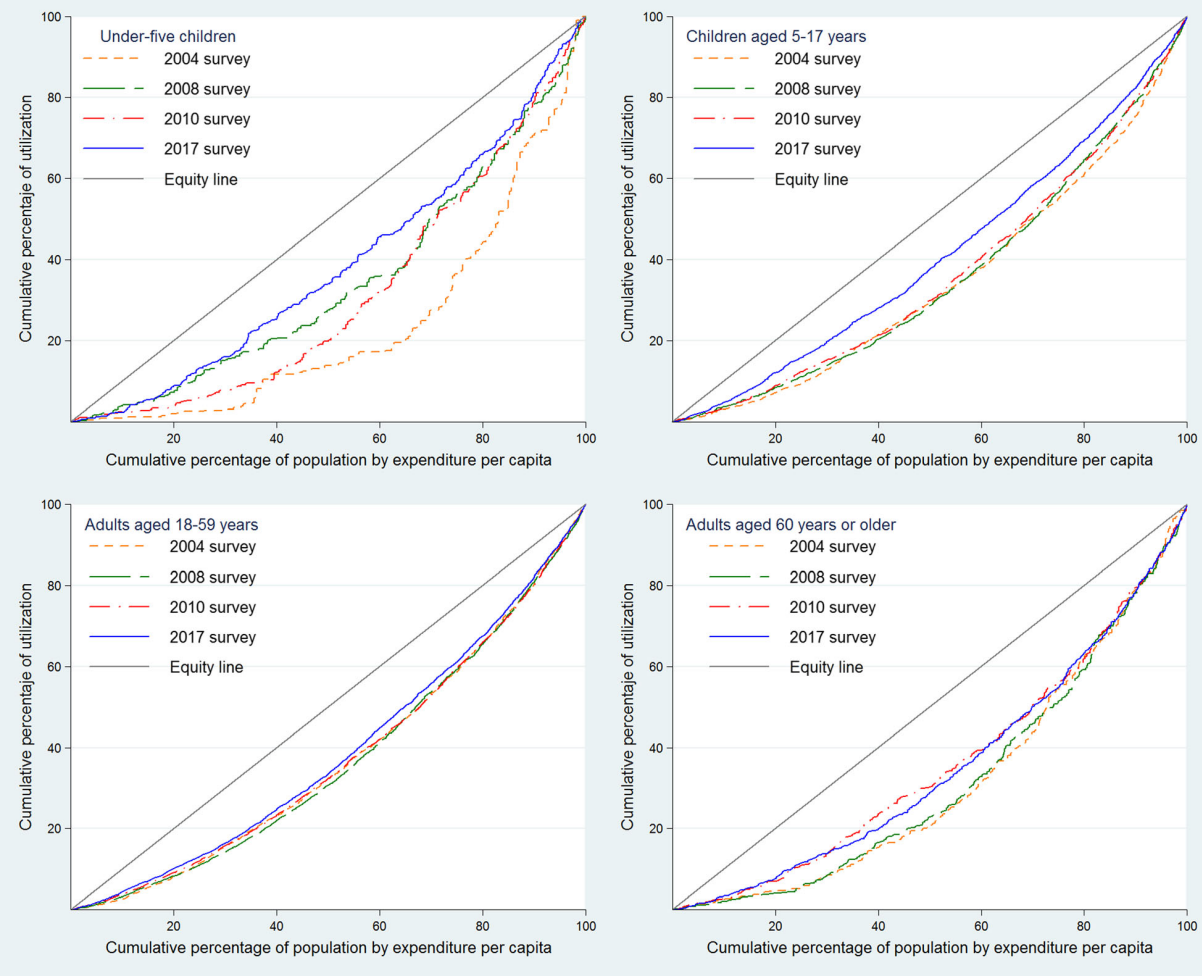
Concentrations curves for access to oral health services by age group. Peru, 2004, 2008, 2010, and 2017**

Regarding CCs for the general population, all curves were placed below the equity line, meaning the dental health services utilization would be concentrated towards the wealthier population (Fig. 1), with a 2017 CCs dominance with respect to the year 2004 and 2010 (Table 3). Age group, area, and natural region-specific CCs were all also placed below the equity line (Fig. 2). Overall, during the first study period, 2004–2008, there was no dominance between CCs excepting rural and coast region, where a slight approach in the equity line is appreciated during 2008 with respect to the 2004 CC (Fig. 1 and Table 3). Conversely, during the 2010–2017 period, 2017’s CC approached the equity line in rural population and Andean and Jungle regions (Fig. 1). Furthermore, during 2017, CCs for less than 5 years and 5–17 years old age group were closer to equity line than the prior from 2010 (Fig. 2).

**Table 3:** Test of dominance between concentration curves of use of dental services, Peru 2004–2017

Population group	Curves of comparation	Significance level	Rule	Test of Dominance (MCA Rule)
General population	2004–2008	0.05	MCA	Non-dominance
	2010–2017	0.05	MCA	2017 dominates 2010
	2004–2017	0.05	MCA	2017 dominates 2004
Under-five children	2004–2008	0.05	MCA	2008 dominates 2004
	2010–2017	0.05	MCA	2017 dominates 2010
	2004–2017	0.05	MCA	2017 dominates 2004
Children aged 5–17 years	2004–2008	0.05	MCA	Non-dominance
	2010–2017	0.05	MCA	2017 dominates 2010
	2004–2017	0.05	MCA	2017 dominates 2004
Adults aged 18–59 years	2004–2008	0.05	MCA	Non-dominance
	2010–2017	0.05	MCA	Non-dominance
	2004–2017	0.05	MCA	2017 dominates 2004
Adults aged 60 years or older	2004–2008	0.05	MCA	Non-dominance
	2010–2017	0.05	MCA	Non-dominance
	2004–2017	0.05	MCA	2017 dominates 2004
Urban	2004–2008	0.05	MCA	Non-dominance
	2010–2017	0.05	MCA	2017 dominates 2010
	2004–2017	0.05	MCA	2017 dominates 2004
Rural	2004–2008	0.05	MCA	2008 dominates 2004
	2010–2017	0.05	MCA	2017 dominates 2010
	2004–2017	0.05	MCA	2017 dominates 2004
Coast	2004–2008	0.05	MCA	2008 dominates 2004
	2010–2017	0.05	MCA	Non-dominance
	2004–2017	0.05	MCA	2017 dominates 2004
Andean	2004–2008	0.05	MCA	Non-dominance
	2010–2017	0.05	MCA	2017 dominates 2010
	2004–2017	0.05	MCA	2017 dominates 2004
Jungle	2004–2008	0.05	MCA	Non-dominance
	2010–2017	0.05	MCA	2017 dominates 2010
	2004–2017	0.05	MCA	2017 dominates 2004

*MCA* Multiple comparison approach

Table 4 shows that between 2004 and 2008 CIs were all greater than zero, defining a wealth-concentrated oral health services utilization. CIs among 2004 less than 5 years (0.0531) and 2008 elderlies (0.366) presented the highest values for each year. The only statistically significant declines in the CIs on this period happened in the less than 5-years of age group and in the rural area. A statistically significant decline in the CIs was detected during the 2010–2017 period. Likewise, statistically significant CIs reductions during the 2010–2017 period were observed that weren’t noted during the 2004–2008 for, general population, 5–17-years-old age group, urban area and Andean and Jungle regions (Table 4). Finally, between 2004 and 2017, the bigger declines in dental health services utilization occurred in the less than 5-year age group (0.295 reductions), Andean region (0.144 reductions) and rural areas (0.132 reductions).

**Table 4:** Summary of results of changes in Concentration Index

Type of health service/Population	Changes in CI 2004–2008			Changes in CI 2010–2017			Change in CI 2004–2017
	CI 2004	CI 2008	Diference of Conc. Index	CI 2010	CI 2017	Diference of Conc. Index	Diference of Conc. Index
Use of dental services							
General population	0.296	0.287	-0.0085	0.264	0.210	-0.0539*	− 0.0855*
Under-five children	0.531	0.312	−0.2187*	0.370	0.236	− 0.1352*	− 0.295*
Children aged 5–17 years	0.308	0.292	−0.0164	0.277	0.185	−0.0918*	−0.123*
Adults aged 18–59 years	0.256	0.265	0.0091	0.252	0.225	−0.0267	−0.030
Adults aged 60 years or older	0.370	0.366	−0.00367	0.285	0.298	0.0129	−0.072
Urban	0.225	0.218	−0.0064	0.209	0.178	−0.0317*	− 0.047*
Rural	0.306	0.239	−0.0663*	0.224	0.174	− 0.0500*	− 0.132*
Coast	0.259	0.232	−0.0261	0.229	0.205	−0.0231	−0.053*
Andean	0.375	0.360	−0.0144	0.310	0.230	−0.0800*	− 0.144*
Jungle	0.311	0.297	−0.0136	0.309	0.249	−0.0601*	− 0.061

*p value < 0.05

*CI* Concentration Index

Z stat for differences

## Discussion

Health access’ equity is a major goal in the global movement for universal health coverage and was one of the main targets of the AUS social reform in Peru during the last decade. The present study confirmed that between 2004 and 2017 dental health services utilization increased among the Peruvian population and the health inequities declined, concurrently with the implementation of the AUS. Notwithstanding, health services utilization still distributes in favor of wealthier populations when analyzed by age group, residence area, and natural region.

Our study contributes evidence on the mechanisms of how AUS reforms can influence the access of all individuals and communities to the comprehensive health services they need and highlights the need to assess progress towards universal health coverage that includes equity measures.

The AUS includes preventive and recuperative dental health-related services for all life stages (e.g. cavities, pulpitis, and gingivitis). This aspect could be one explanation for the phenomenon found related to the increased use of oral health services. Similar situations have been reported in countries like China, where after the 2007 implementation of urban children and unemployed adult’s Basic Medical insurance**,** major improvements in health insurance inequality were shown with an increase in the health insured population coverage from 49,7% in 2006 to 90,8% in 2009. This increase would enable health services utilization [16]. Likewise, an increase in dental health services utilization was found in Chile after a major health reform with a decrease in socioeconomic inequity [14]. Nevertheless, universal insurance implementation isn’t by itself able to eradicate health services utilization inequality, without the aid of determinant matters like income quintile, literacy, natural region residency, age and type of health insurance [19]. It is also essential to concentrate strategies on groups that have greater needs for the use of services. Additionally, international experiences where the restriction of prior benefits in dental health care shows us that the failure to provide preventive services triggers a higher health cost to the system, as in the case of Illinois, USA, where dental insurance was restricted to emergency treatment only, resulting in an increase in the total cost of dental health of $1.6 million. In that sense, health coverage should mandatorily include basic preventive oral health services [20].

In general, the concentration in oral health services utilization was found around wealthy individuals in Peru, compared with people with lower expenditure capacity. Similar scenarios were found in other countries around the region, like Brazil, where the higher income individuals agglomerate the biggest dental health utilization proportion [21, 22]. These findings could be explained due to several reasons, like the fact that wealthier populations can afford higher cost and quality private services which aren’t offered in public health facilities. On the other hand, low resource populations can access public services, yet they rarely are able to attend because of time restrictions when they have informal jobs or previous failed treatment experiences. Additionally, these public health services facilities often offer limited and low-quality services [23–26].

Inequality in the use of oral health services in Peru, affecting mainly economically disadvantaged groups, could favor the development of diseases such as cavities which are the most frequent oral health problem. It is important to know that people with a lower socioeconomic level are a group in greater need of oral health care as they are more vulnerable to these diseases [27]. Oro-dental diseases’ effects such as pain, suffering, functional decline and decreased quality of life are significant and costly. Estimations report that dental health treatments represent between 5 and 10% sanitary expenditure in developed countries and exceed the spending capacity of many developing countries. As with other chronic diseases, dental diseases exhibit a substantial social gradient, creating unacceptable inequities. It is unfair that people from disadvantaged backgrounds experience high levels of dental diseases. The negative consequences of oral diseases such as poorer school performance and reduced employment opportunities, low self-esteem and social isolation all contribute to wider health inequalities in society [28].

At the national level, several successful preventive promotional interventions with vulnerable communities exist, which reported several improvements in cavities control, number of obturations made and general oral hygiene [29]. In addition, similar cases have been reported internationally, where preventive treatment access in low resource children populations through interdisciplinary collaboration, improved cavities rates and odontologist visits [30]. Furthermore, implementation of preventive oral health programs for children represents big savings to the health system in oral treatments that double the implementation costs [31].

Regarding the inequality in health access across urban populations reported in our study, several reports show a similar trend, confirming that this is a global problem [32]. Several strategies are being developed to evaluate not only oral health inequalities between urban and rural settings but also inside the urban setting, knowing that they usually are heterogeneous with higher risk groups for dental problems [33]. On the other side, it has been demonstrated that several existing community preventive oral health strategies could be rolled out in hard to reach populations, such as rural areas, where properly focalized would improve oral diseases rates [31, 34].

Generally, all age groups presented a reduction of the oral health services utilization inequality, excepting the elderly (60 years or older) population, which presented an increase in inequality in favor of the wealthier population. In this regard, we can mention that in this specific case, AUS doesn’t contemplate oral rehabilitation procedures for the elderly population [11], the ones who suffer partial or complete edentulism as one of the main health problems. In that sense, there are only a few oral health rehabilitation programs for elderly people, such as the national plan “Smile Again” (“*Vuelve a sonreír*”), but this initiative is focused on poor and extremely poor populations and it worked until 2016 [35]. The period of non-functioning of the program in 2017 may be affecting the increment of the inequality gap. Likewise, in Chile, the age group with the biggest inequalities in oral health services utilization was the elderlies, and the lowest inequality was found among the underage population [36], very similar to our findings. Studying oral health access by age group is very important, as it allows interventions to be focused on the most needed groups with the use of directed and focalized strategies.

Given these variations in the inequality of oral health services access in the period of our study, it is important to put in context the implementation of various oral health programs in recent decades in Peru. The National Concerted Health Plan (2007) included as one of its priority goals the improvement of oral health by reducing the dental caries rates in children and improving the preventive care coverage of pregnant women living in poverty. All these activities were planned through the expansion of the offer of dental services in the 10 poorest regions of Peru and the deployment of sanitary teams in remote populations [37]. That same year, the “Oral Health Strategy” of the General People’s Directorate of Health was created, establishing within its functions the activities of prevention, promotion, and recovery of oral health. Likewise, in its 2012 restructuration, the National Plan “Smile Again” was implemented, with the objective of rehabilitating the edentulous elderly [38].

Regarding the scope of the study, there are limitations associated with a secondary analysis of a survey that presents data obtained by self-reporting respondents, where there may not be precision in the data or recall bias. In some areas of the country, the respondents could have confounded the survey evaluators as the staff of a social programme, and due to ignorance or fear of losing some kind of benefit, could have provided incorrect data. In addition, there is a three-month window of time for this study that could leave out people who reported oral health service use from previous months. However, the results found are valid and provide important information for the planning and administration of health services as they come from a population survey, which is carried out annually and is designed to measure the living conditions of the Peruvian population.

## Conclusions

Oral health services utilization increased in Peru and inequality decreased during the 2004–2017 period, concurrently with the implementation of the AUS. However, the use of these services continues to be distributed in favor of the richest populations. New oral health strategies and programs need to be introduced to the Peruvian population in order to close the gap in the use of oral health services, currently driven by economic inequalities.

## Abbreviations

AUS: Universal Health Assurance (Aseguramiento Universal en Salud, in Spanish)
CC: Concentration curve
CI: Concentration index
ENAHO: National Household Survey on Living Conditions and Poverty (Encuesta Nacional de Hogares sobre Condiciones de Vida y Pobreza, in Spanish)
MINSA: Ministry of Health of Peru. (Ministerio de Salud, in Spanish)
